# Toll-like receptor (TLRs) agonists and antagonists for COVID-19 treatments

**DOI:** 10.3389/fphar.2022.989664

**Published:** 2022-09-07

**Authors:** Zhi-Mei Liu, Ming-Hui Yang, Kun Yu, Zheng-Xing Lian, Shou-Long Deng

**Affiliations:** ^1^ Beijing Key Laboratory for Animal Genetic Improvement, National Engineering Laboratory for Animal Breeding, Key Laboratory of Animal Genetics and Breeding of the Ministry of Agriculture, College of Animal Science and Technology, China Agricultural University, Beijing, China; ^2^ Key Laboratory of Molecular Medicine and Biotherapy, Key Laboratory of Medical Molecule Science and Pharmaceutics Engineering, Advanced Research Institute of Multidisciplinary Sciences, Beijing Institute of Technology, Beijing, China; ^3^ NHC Key Laboratory of Human Disease Comparative Medicine, Institute of Laboratory Animal Sciences, Chinese Academy of Medical Sciences and Comparative Medicine Center, Peking Union Medical College, Beijing, China

**Keywords:** SARS-CoV-2, TLRs agonists, TLRs antagonists, COVID-19 infection, innate immunity

## Abstract

Severe acute respiratory syndrome coronavirus type 2 (SARS-CoV-2) rapidly infects humans and animals which make coronavirus disease 2019 (COVID-19) a grievous epidemic worldwide which broke out in 2020. According to data analysis of the other coronavirus family, for instance severe acute respiratory syndrome SARS coronavirus (SARS-CoV), can provide experience for the mutation of SARS-CoV-2 and the prevention and treatment of COVID-19. Toll-like receptors (TLRs) as a pattern recognition receptor (PRRs), have an indispensable function in identifying the invader even activate the innate immune system. It is possible for organism to activate different TLR pathways which leads to secretion of proinflammatory cytokines such as Interleukin 1 (IL-1), Interleukin 6 (IL-6), Tumor necrosis factor α (TNFα) and type Ⅰ interferon. As a component of non-specific immunity, TLRs pathway may participate in the SARS-CoV-2 pathogenic processes, due to previous works have proved that TLRs are involved in the invasion and infection of SARS-CoV and MERS to varying degrees. Different TLR, such as TLR2, TLR4, TLR7, TLR8 and TLR9 probably have a double-sided in COVID-19 infection. Therefore, it is of great significance for a correctly acknowledging how TLR take part in the SARS-CoV-2 pathogenic processes, which will be the development of treatment and prevention strategies.

## Introduction

Since 2020, SARS-CoV-2 led to a dramatic mass epidemic of COVID-19 worldwide, infecting hundreds of millions of people in the worldwide which generated a disease disaster. COVID-19, not only cause severe lung inflammation in frail patients, but also cause damage to the heart, kidneys and other organs, and ultimately lead to death of the patient (Huang et al., 2020).

The immune pathogenesis of novel Coronavirus is not clear. Furthermore, there are other new variants virus, which may lead to available vaccines failure (Harvey et al., 2021). TLRs are principally divided into TLRs in human cells (TLR1-TLR10) and TLRs in mouse cells (TLR1-TLR9 and TLR11-TLR13), both of which are encoded by a large gene family (Kawasaki and Kawai, 2014). According to the localization of each TLRs, TLRs can be roughly divided into two categories. One is located on the cell surface, including TLRs 1, 2, 4–6, and 10. The other type of TLRs locates on the endosomal membrane of cells, including TLRs three and 7–9 (O'Neill et al., 2013). TLR reaction could eventually through different signaling pathways induce interferon (IFN), cytokines and chemokines, thereby refrain the pathogen to the body’s further infection, and trigger the corresponding pathogen of adaptive immune response (Takeuchi and Akira, 2010; Kawai and Akira, 2011). Compared with chemotherapy drugs, immunotherapy targeting TLRs is capable to inhibit viral infection and reduce cellular inflammatory cytokines secretion through immune regulation, and can as well as be used to improve COVID-19 vaccination strategy as vaccine adjuvant (Choudhury et al., 2021). Inappropriate use of TLRs antagonists in the treatment of COVID-19 may lead to a decrease in IFN without inhibiting the virus. Therefore, the dosage and duration of TLRs antagonists should be further tested in the clinical stage (Patra et al., 2021; Patra et al., 2021).

Different TLRs, such as TLR2, TLR4, TLR7, TLR8, and TLR9, may have a double-sided effect in COVID-19 infection. Therefore, it is of the essence to correctly understand whether different TLRs are involved in the infection process of SARS-CoV-2. Many viruses enter the body and are recognized by TLRs to activate the innate immune system, which gives the body the possibility of clearing the virus or suppressing its proliferation, although persistent inflammation can cause tissue damage. Cellular endosomal TLR3, TLR7, TLR8 and TLR9 have the ability to identify not-self nucleic acid. Although SARS-CoV-2 is a single-stranded RNA virus, it produces double-stranded RNA during viral replication, which gives the virus the possibility to be recognized and bound by different TLRs (Horova et al., 2021; Li et al., 2021). In our review, the SARS-CoV-2 invasion and pathogenic molecular mechanisms, the roles of different TLRs in SARS-CoV-2 infection, and whether agonists and antagonists of TLR can be used as potential therapeutic drugs for COVID-19 will be discussed.

## SARS-CoV-2

After two outbreaks of SARS-CoV and MERS-CoV, SARS-CoV-2 broke out and spread rapidly which is the third zoonotic virus belonging to the same coronavirus family (Guarner, 2020). Like SARS-CoV, novel SARS-CoV-2 contains about 30 kilobases of single-stranded RNA, which encodes a variety of component proteins and envelope protein in the outer layer (Lu et al., 2020). The genomes of SARS-CoV-2 and SARS-CoV are highly similar, more than 80% (Wu et al., 2020). About the structural proteins, there are spike protein (S), membrane protein (M), envelope protein (E), and nucleocapsid protein (N) (Ahmed et al., 2020). Coronaviruses are roughly categorized into four genera, including α-CoV, β-CoV, γ-CoV, and δ-CoV (Perlman and Netland, 2009; Chan et al., 2020). SARS-CoV-2 essentially shares a set of similar characteristics of the B coronavirus, which is extremely prone to mutate into different mutants and can infect different hosts at the same time (Ye et al., 2020) which is extremely similar to SARS-CoV. The SARS-CoV-2 envelope (E) protein has the function of forming viral pore protein on the membrane and promoting virus maturation. Membrane protein (M) takes part in the assembly of SARS-CoV-2 morphogenesis and virus replication by interacting synergistically with other compositional proteins.

Spike proteins (S), a kind of glycoprotein naked in surface, is essential to attach, fuse, and invade organism for SARS-CoV-2 (Wrapp et al., 2020). The common feature of SARS-CoV-2 and SARS-CoV invasion is that they both bind angiotensin converting enzyme 2 (ACE 2) on human target cells (such as nasal and bronchial epithelial cells and type II alveolar cells in the lungs) via external surface spiking S protein. SARS-CoV-2 also infects a wide range of cells with ACE2 receptors distributed externally in the lung, and ACE2 receptors have also been found on tissues of the stomach, intestinal, cardiovascular, and central nervous system tissues (Wadman et al., 2020). The SARS-CoV-2 S protein is also primed via host cell-associated transmembrane protease serine 2 (TMPRSS2). TMPRSS2 cleaves S protein and activates S2 domain, promoting the fusion of SARS-CoV-2 envelope with host cell membrane to complete invasion (Hoffmann et al., 2020).

## TLRs

The human immune response contains innate immune and adaptive immune, which are the protective lines for preventing the human body from pathogen infection. When a pathogen invades the body, it triggers the first line of defense, the innate immune system.

The pivotal innate immune sensing receptors can not only recognize different molecular signatures of foreign antigens called pathogen-associated molecular patterns (PAMPs), but also molecules released from organism damaged cells, named as damage-associated molecules patterns (DAMPs). PRRs sense ligands and induce the production of cytokines, type I IFN, then trigger a signal downstream of the receptor which innate following immune responses, including antigen-specific adaptive immune responses and inflammatory responses (Janeway and Medzhitov, 2002).

These responses are critical for the clearance of infected microorganisms and subsequent instructions for antigen-specific adaptive immune responses. These interferons (IFNs) are antiviral molecules and inhibit the replication efficiency of the virus (Mogensen and Paludan, 2005). Specialized B lymphocytes and T lymphocytes participate in the adaptive immune process of the body, and can produce specific antibodies against antigens, and form memory immunity when the next antigen invasions (Mogensen and Paludan, 2005).

TLRs was first identified, and categorized as TLR1-TLR13 (Takeuchi and Akira, 2010). Each TLR consists of an extracellular domain that mediates PAMPs recognition of Leucine-rich repeats (LRRs), a transmembrane domain, and a cytoplasmic Toll/IL-1 receptor (TIR) domain that initiates downstream signaling transduction (Kawasaki and Kawai, 2014). The extracellular domain shows a horseshoe-shaped structure in which TLRs interact with their respective PAMPs or DAMPs to form homomorphic or heteromorphic dimers accompanied by coreceptors or helper molecules (Botos et al., 2011; Behzadi et al., 2021).

TLRs are expressed on both innate immune cells and non-immune cells. TLRs are widely distributed and participate in extensive innate immunity of the body. TLRs can be divided into cellular surface TLRs and intracellular TLRs according to different distribution locations. TLRS distributed on cell surface included TLR1, TLR2, TLR4, TLR5, TLR6 and TLR10, while TLR3, TLR4, TLR7-9 and TLR11-13 were distributed on the membrane of intracellular endosomes (Kawai and Akira, 2011; Kawasaki and Kawai, 2014). Intracellular regions mainly consist of endoplasmic reticulum (ER), endosomes, lysosomes, or endo-lysosomes, which can provide the cell with a comprehensive immune defense and recognize different PAMPs such as lipids, lipoproteins and nucleic acids (Kawasaki and Kawai, 2014).

**FIGURE 1:**
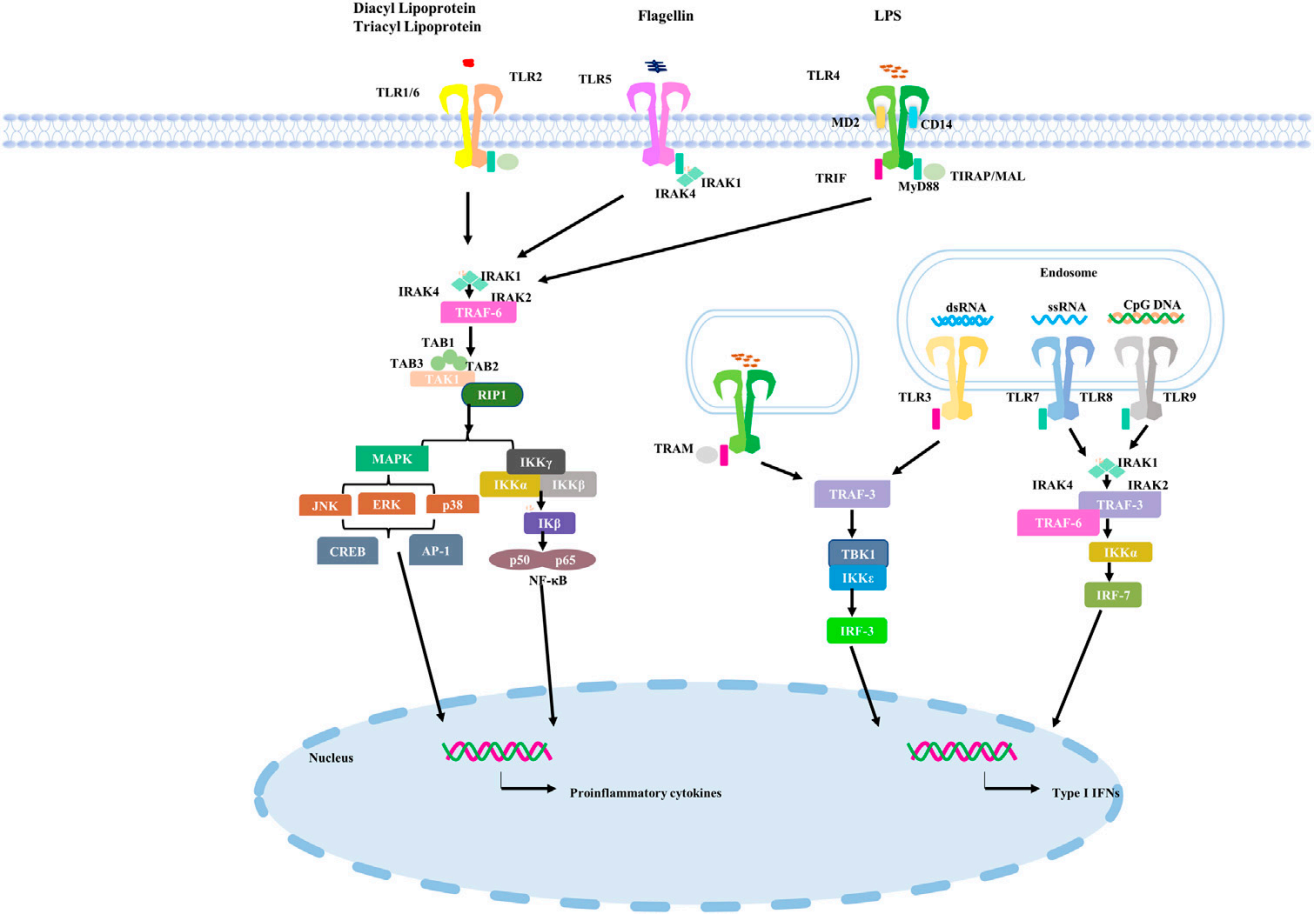
TLRs signaling pathway in the human.

TLRs located on the cell surface primarily perceives microorganism for instance microbial lipids and lipoproteins. TLR4 homologous dimer senses bacterial lipopolysaccharides (LPS) of Gram-negative bacteria, heat shock proteins and some components of viruses for example Respiratory syncytial virus (RSV) as the ligands (Akira and Takeda, 2004; Akira et al., 2006; O'Neill et al., 2013). In the TLRs family, the subcellular localization of TLR4 is unique and can be found on the cell surface or in vesicles of endocytic bodies, which also means that it has special functions (Pelka et al., 2018). TLR2 usually acts as a heterodimer with TLR1 or TLR6 to recognize teichoic acids, lipopeptides, peptidoglycans and yeast glycans (Kawai and Akira, 2011). The intracellular TLRs ultimately contain TLR3、TLR7、TLR8 and TLR9. For example, TLR3 recognizes viral double-stranded RNA (dsRNA) during RNA virus replication in infected cells (Leonard et al., 2008; Cheng et al., 2011; Zhang et al., 2018). It recognizes the ribose-phosphate backbone rather than substrate sequences (Liu et al., 2008). TLR7 principally expresses in plasmacytoid dendritic cells (pDCs) and basically senses and binds to single stranded (ss)RNA from the virus. RNA of viral and bacterial are the primary ligands to TLR8 (Guiducci et al., 2013). TLR9 differs from TLR7 and TLR8 in that it mainly uses bacterial or viral DNA sequences with abundant unmethylated CPG-DNA motifs as recognition ligands (Coban et al., 2010). TLRs signaling pathway in the human is shown in Figure 1.

## TLRs sensing of SARS-CoV-2

TLRs first bind to ligands and then recruit adaptor proteins, comprising myeloid differentiation primary response 88 (MyD88) and TIR domains (TRIF) containing adapter-induced IFN-β, which activate different downstream immune molecular pathways (Kasuga et al., 2021). The activation of TLRs may have a double-sided effect. Moderate activation can provide protection for the body from the invasion of antigens. Excessive TLRs activation can lead to severe inflammatory responses that can lead to damage and even death (Yokota et al., 2010; Harsini et al., 2014; Dajon et al., 2017; Ebermeyer et al., 2021; Zheng et al., 2021), which has also been observed in cases of SARS-CoV-2 infection (Zheng et al., 2021).

### TLR2

TLR2, a receptor in cell surface, can recognize various ligands from viruses, bacteria, fungi and parasites (Oliveira-Nascimento et al., 2012). TLR2 acts as heterodimer and uses MyD88 pathway for signaling transduction. Although the detailed mechanism of whether TLR2 is involved in the immune response of patients infected with SARS-CoV-2 has not been clarified, a recent study suggested that it is possible for TLR2 sensing SARS-CoV-2 E protein (Sariol and Perlman, 2021; Zheng et al., 2021).

A study has shown that TLR2 can sense and bind SARS-CoV-2 E protein and further induce the production of proinflammatory cytokines during coronavirus infection. Inhibition of TLR2 and its downstream signaling pathways in the body can prevent and control the occurrence of COVID-19.

Zheng and colleagues used public data sets to analyze t the expression of TLRs and downstream adaptor proteins in COVID-19 patients with varying degrees of disease progression revealed that E protein can activate TLR2 and downstream immune signaling pathways.

In addition, SARS-CoV-2 E protein can induce TLR2-related inflammatory responses in experimental group mice, while TLR2 inhibitors can protect mice from mortality caused by SARS-CoV-2 infection. These data indicate that TLR2 can recognize and bind SARS-CoV-2 E protein to activate downstream TLR2 signaling pathway and achieve antiviral immune response (Zheng et al., 2021). About a further research, recombinant SARS-CoV-2 S protein also activated the TLR2-mediated NF-κB pathway to activate inflammatory factors release in human immune cells. This result requires further experimental study (Jung and Lee, 2021).

### TLR2 agonists

TLR2, highly expressed in neutrophils, macrophages, and dendritic cells, recognizes peptidoglycan and lipoteichoic acid components in Gram-positive bacteria cell walls, and subsequently forms heterodimer complexes with TLR1 to activate relevant downstream signaling pathways and ultimately induces NF-κB release which is able to induce oxidative stress and contribute towards the destruction of pathogens (Li et al., 2019). Pam3CSK4, TLR1/2 agonist, has been studied in combination with protein adjuvants as alternative vaccine for SARS-CoV-2. Studies about the vaccine showed that Pam3CSK4 protects organism against SARS-CoV-2 virus (Zhou et al., 2022).

### TLR1/2 antagonists

Overexpression of TLR1/2 may promote the excessive cellular inflammatory response induced by SARS-CoV-2, hence small molecule inhibitors of TLR2 may be a potential agentia to prevent strong and excessive inflammatory response (Zheng et al., 2021). A study of experimental TLR2 inhibitor oxPAPC in mice expressing human ACE2 showed that oxPAPC significantly reduced the release of inflammatory cytokines as well chemokines even during the process of viral invasion (Zheng et al., 2021). Targeting TLR2 with antagonists significantly reduce death rates of SARS-CoV-2-infected individual compared with control-treated mice, suggesting that TLR2 probably participate in SARS-CoV-2-infected process. Further, this study has implications for the COVID-19treatment and prevention: applying TLR2 antagonists such as oxPAPC in patients with outbreaks of excessive inflammation may reduce the inflammatory response and possibly improve the cure rates of patients (Zheng et al., 2021).

### TLR4

TLR4 exists on both cell surface and endosomal membrane. TLR4 at different locations can trigger different downstream signaling pathways through different adaptors. (Kuzmich et al., 2017). Recently, two groups of studies have demonstrated SARS-CoV-2 S protein causes monocytes and macrophages to produce pro-inflammatory cytokines through TLR4-mediated related signaling pathways (Shirato and Kizaki, 2021; Zhao et al., 2021). A study suggested that S1 subunit (residue 16–671) induced the NF-κB and mitogen-activated protein kinase pathways (MAPK), afterwards macrophages produce the proinflammatory cytokines (Shirato and Kizaki, 2021).

Intracellular viral sensors play master roles in identifying SARS-CoV-2, leading to effective type I IFNs and cytokine responses. Moreover, it is possible for SARS-CoV-2 to escape TLR perception, which may account that, the body has not yet established effective immunity against the virus at the initial stage of SARS-CoV-2 invasion (van der Donk et al., 2022). This leads to inefficient DCs activation and subsequent abnormal inflammatory responses.

TLR4 is important in inducing host immune responses against infectious diseases, such as bacterial, fungal and viral infections and malaria (Deng et al., 2012; Mukherjee et al., 2016). Nevertheless, overstimulation induced by TLR4 can lead to excessive inflammation. Compared with other TLRs, the protein-protein interaction between TLR4 and SARS-CoV-2 spike glycoprotein is the strongest (Choudhury and Mukherjee, 2020). In addition, SARS-CoV-2 strongly induces the expression of interferon stimulating gene (ISG) in the context of respiratory immunopathogenesis (Zhou et al., 2020).

COVID-19 causes acute lung injury (ALI) and acute respiratory distress syndrome (ARDS), which leads to lung failure and subsequent death (Shereen et al., 2020). Clinical results showed that the main cause of death caused by SARS-CoV-2 was respiratory failure (Ruan et al., 2020; Zhang et al., 2020). One study also found that cell surface TLRs have the ability to more easily recognize and bind SARS-CoV-2 to activate downstream signal transduction pathways and trigger the secretion of inflammation-related factors (Bhattacharya et al., 2020).

TLR4 signaling transduction pathways are divided into MyD88-dependent and non-MyD88-dependent pathways (Kawasaki and Kawai, 2014). When the TIR domain differentially binds to the MyD88 adaptor protein, the MyD88-dependent pathway is initiated. MyD88 then recruits and then activates the adaptor proteins IRAK4 and IRAK1 receptor-associated kinases, followed by TRAF6, transforming growth factor-β-activated kinase 1 (TAK1) and TAK1-binding protein 2 (TAB2) (Jiang et al., 2002; Kollewe et al., 2004; Chen, 2012; Ajibade et al., 2013). Ultimately, TAK1 triggers subsequent activation of IKKs or MAPK. Furthermore, transcription of IL-12, IL-6 and TNF-α is induced in the nucleus by ERK1/2, P38 and JNK1/2 pathways or by dimerization of the P65 and P50 subunits of NF-κB into the nucleus (Akira and Takeda, 2004). Withal, when TLR4 recruits TRIF and TRAM, it results in recruitment of kinases TBK1 and IKKε (Akira and Takeda, 2004; Satoh and Akira, 2016). The phosphorylation of these regulatory transcription factors, IRF3, producing type I IFNs (Satoh and Akira, 2016). Type I IFNs (IFN-α and β), as antiviral cytokines, functions as the first line to defense against the pathogen invasion (Samuel, 2001).

Evidence from *in vivo* studies suggests that MyD88^−/−^ and TRIF^−/−^ mice infected with SARS-CoV exhibit higher mortality, weight loss, and high viral load. Hence, MyD88 and TRIF are necessary for protection against fatal infection from SARS-CoV (Sheahan et al., 2008; Totura et al., 2015). A balance between two kinds of signaling pathways is necessary for producing appropriate innate immune response. Another earlier study showed that SARS-CoV M protein, a novel cytosolic PAMPs, promotes IFN-β induction through a TLR-associated TRAF3-independent mechanisms (Wang and Liu, 2016). Therefore, the SARS-CoV-2 M protein may induce TLR4-mediated TRAF3-independent IFN-β production intracellularly.

SARS-CoV-2 infects some organs that express TLR4 with relatively low ACE2 expression, such as skin fibroblasts, resulting in complications (Bhattacharyya et al., 2017; Fu et al., 2020). Meanwhile, it has been covered that TLR4 as well its downstream signaling mediators are notably upregulated in peripheral blood monocytes in COVID-19 patients compared to control individuals (Sohn et al., 2020). Inflammatory molecules are detected to be raised including CD14, MyD88, TIRAP, TRAF6, IRAK1 and TRIF. A study reported increased TLR4 expression in myocardium of COVID-19 patients (Kogan et al., 2020) resembles the significant increase of TLR4 expression in bacterial sepsis myocardium (Rameshrad et al., 2015; Khodir et al., 2020). It indicates that TLR4 may sense SARS-CoV-2 and be activated to increase expression. According to Aboudounya, SARS-CoV-2 is also a coronavirus, presumably it can be inferred that SARS-CoV-2 directly activates TLR4 by binding S protein to TLR4 (Aboudounya and Heads, 2021).

Cytokine storm syndrome induced by SARS-CoV-2 frequently leads to illness progression and death in COVID-19 patients (Ruan et al., 2020). Previous researches suggested that SARS-CoV-2 may cause cytokine storm syndrome by destroying the balance between two downstream signaling pathways, resulting in abnormal downstream TLR4 signaling (Giamarellos-Bourboulis et al., 2020; Lucas et al., 2020). This result reported that IL-1β, IL-6, IL-18, and TNF-α, downstream of TLR4-MyD88-dependent pathway were higher in severe COVID-19 patients with cytokine storms (Giamarellos-Bourboulis et al., 2020; Lucas et al., 2020).

### TLR4 agonists

Previously, inulin acetate (InAc) is from a plant polysaccharide inulin which can be capable of activating TLR4 and triggering a powerful body’s immune response when it is used as a vaccine adjuvant (Kumar et al., 2017). Previous studies have shown that InAc has the ability to activate TLR4, increasing TLR4 expression on a variety of APCs cells and triggering downstream signal transduction pathways (Kumar et al., 2016; Kumar et al., 2017; Rajput et al., 2018).

SARS-CoV-2 is known to infect the human body mainly through a variety of mucous membranes, such as respiratory mucosa and eye mucosa. Therefore, forming a strong immune response in the mucous membrane can make a timely response to the virus invasion and prevent the virus invasion. A recent study described nanoparticles prepared using InAc (InAc-NPs), a TLR4 agonist, as an intranasal vaccine delivery system that elicits a strong enough immune response at mucosal sites and throughout the body (Bakkari et al., 2021). Taken together, InAc-NPs has the ability to maintain an effective memory immune response after an immune response, and is a potential mucosal inoculated intranasal agent for COVID-19 immunotherapy using TLR4 activator combined with adjuvant, which can inhibit the early invasion of SARS-CoV-2 infection. Additionally, glycopyranosyl lipid adjuvant (GLA) formulated with SE and mixed with antigen also activate TLR4 and induces Th1 immune response in mice (Raman et al., 2009; Bertholet et al., 2010; Raman et al., 2010; Dubois et al., 2016).

### TLR4 antagonists

Studies have shown that treatment with appropriate TLR4 antagonists late in the development of COVID-19 patients can inhibit excessive TLR4 expression and thus inhibit inflammatory cytokine storms (Mukherjee, 2022). To date, the first natural TLR4 antagonist in history was identified from the non-pathogenic photosynthetic Gram-negative bacterium Erythrobacterium globosus. The bacterium naturally produces a non-toxic lipopolysaccharide called RsDPLA, which has the ability to compete with toxic lipopolysaccharides for TLR4 binding sites on cell surfaces (Anwar et al., 2015). In subsequent research and exploration of disease treatment, ERdolian (E5564) and E5531, TLR4 antagonists, were homeopathy produced and modified and synthesized from the original design of RsDPLA (Kawata et al., 1999; Barochia et al., 2011). E5531 is the first batch of lipid A analogue that was synthesized for a septic shock treatment (Kobayashi et al., 1998). The E5564 is an improved product based on the E5531 and has a stronger anti-endotoxin effect (Mullarkey et al., 2003).

Glycyrrhizin is an active ingredient and has potential effect to be used to control COVID-19 because of its dual antiviral action and TLR4 antagonism (Aboudounya and Heads, 2021). It has anti-inflammatory capacity by down-regulating HMGB1 mediated inflammation and by antagonizing TLR4 (Andersson et al., 2020; Murck, 2020). It is metabolized in the human gut (Murck, 2020). It was reported that Glycyrrhizin showed the ability to restrain the replication and resist the invasion of SARS-CoV *in vitro* (Cinatl et al., 2003; Andersson et al., 2020; Murck, 2020). Furthermore, aryl 7-chloroquinolinyl hydrazone (3a-u) is a synthetic anti-inflammatory drug, which can play an anti-inflammatory effect by inhibiting the activation of macrophage downstream inflammatory cytokine secretion pathway after LPS binding to TLR4. Such inhibitors also provide a possible combination regimen for the clinical treatment of COVID-19 patients with severe inflammatory reactions (Debnath et al., 2019).

### TLR3

TLR3 on cell endosomal membrane is transformed from monomer to dimer by binding with dsRNA (Wang et al., 2010) and then activates downstream signal transduction through the adaptor protein TRIF (Piras and Selvarajoo, 2014).

TLR3 recognizes and binds to ligands to promote the formation of TLR3 dimers, then TRIF recruits receptor-interacting protein 1 (RIP1) and tumour necrosis factor receptor-associated factor 3 (TRAF3), which act as a scaffold for bridging TANK Binding Kinase 1 (TBK1) and IκB kinase ε (IKKε), leading to phosphorylation and the activation of the interferon regulatory factor 3 (IRF3) or IRF7 (Kawai and Akira, 2010). And ultimately lead to IFNs and inflammatory cytokine expression. A previous in silico study has shown that TLR3 has the potential to recognize and bind to the mRNA encoding the NSP10 protein of SARS-CoV-2, thereby triggering downstream related pathways (Choudhury et al., 2021). Another study revealed that TLR3-IRF7-mediated deficiency of type I IFNs immune innate response probably bring about high mortality (Zhang et al., 2020). Another study on the role of TLR3 in SARS-CoV-2 infection, using SARS-CoV-2 to infect Calu-3/MRC-5 multicellular spheroids (MTCSs), found a prominent increase in TLR3-associated IRF3 expression during the first 24 h after infection, followed by IL-1α, Il-1β, IL-4, IL-6 and IFN-α and IFN-β (Bortolotti et al., 2021). Subsequent administration of TLR3 inhibitors reduced the levels of these inflammatory cytokines and type I interferons. To sum up, TLR3 is activated within 24 h of SARS-CoV-2 infection, which may produce inflammatory factors in the early stage of virus infection by sensing the dsRNA produced during the replication of SARS-CoV-2, thereby facilitating the subsequent immune response against the virus.

### TLR3 agonists

The small molecule CU-CPT17e (17e) activates TLR3 (Salyer et al., 2016) and can induce the release of cytokines in the body to produce an immune response against antigen invasion (Zhang et al., 2017). IPH-3102 is also a TLR3 activator, an artificially generated dsRNA agent, that specifically activates the TLR3-mediated NF-κB signaling pathway and type I IFNs response (Panter et al., 2009; Pradere et al., 2014).

The TLR3/MDA5 synthetic agonist Poly (I: C) was shown to be effective against lethal doses of SARS-CoV-2 in K18-HACE2 transgenic mice. Early Poly (I: C) treatment accompanied by reduced viral load, prevention of cytokine storms in lung and brain cells, and increased macrophage and NK cell levels which resulted in an 83% survival rate in mice with long-term immunization (Tamir et al., 2022). Therefore, initiating lung innate immunity with Poly (I: C) or similar methods can provide effective and safe protection against SARS-CoV-2 infection.

Other studies have shown that a combination of TLR1/2 and TLR3 agonist (L-PAMPO) can be an effective adjuvant for a SARS-CoV-2 subunit vaccine to activate adequate body immunity against SARS-CoV-2 (Jeong et al., 2021). Experimental results revealed that L-PAMPO combined with SARS-CoV-2 antigen produced a vaccine that elicits a strong immune response in a ferret model and significantly reduces viral load during nose washing.

## TLR7,TLR8,TLR9

In contrast, TLR7 and TLR8 sense ssRNA and chose adapter protein MyD88 to induce the downstream pathway. Recent studies used three-dimensional pulmonary multicellular spheres to determine the functions of TLR3 and TLR7 in the immune response to SARS-CoV-2 infection (Bortolotti et al., 2021). In SARS-CoV-2-infected three-dimensional pulmonary multicellular spheres, the relative expression levels of TLR3 and TLR7 and the production of type I IFNs and pro-inflammatory cytokines were increased.

In addition, TLR7/8 heterodimer can be activated by ssRNA with abundant guanosine (G) and uridine (U) (Li et al., 2013). Another experiment demonstrated that two GU-rich ssRNA sequences in SARS-CoV-2 RNA, called SCV2-RNA, activate TLR7/8 and its downstream MyD88 related pathways in human DCs (Salvi et al., 2021). As well, studies revealed that TLR7 recognizes coronaviruses in a few types of immune cells, such as cDCs and pDCs, and triggers the production of type I interferons and pro-inflammatory cytokines (Cervantes-Barragan et al., 2007). In addition, experimental results suggest that TLR7 is involved in regulating IFN production during MERS-CoV infection (Scheuplein et al., 2015). Another therapeutic study found that natural mutations in the TLR7 gene encoding expression can actually affect disease severity and mortality in young sufferers with COVID-19, thus demonstrating that TLR7 is involved in SARS-CoV-2 infection and recognizes the virus as a candidate therapeutic target (van der Made et al., 2020; Solanich et al., 2021).

TLR9 has ability to recognize short sequences of CPG-rich DNA from bacterial, viral and mitochondrial DNA (mtDNA) (Zhang et al., 2010). Previous studies indicated that CpG, a key TLR9 recognition element, is abundant in the SARS-CoV-2 E protein and ORF10 coding regions, which provides the possibility that SARS-CoV-2 can be recognized by TLR9 as a ligand (Digard et al., 2020).

### TLR7/8 agonists

Activation of TLR7 can inhibit viral infection through Th1 immune response, and may also produce bronchiectasis and anti-inflammatory effects (Medzhitov, 2001; Akira and Takeda, 2004). SARS-CoV-2 has more ssRNA motifs that can bind to TLR7 (van der Made et al., 2020; Solanich et al., 2021). TLR8 also recognizes ssRNA viruses such as influenza, human immunodeficiency virus (HIV), hepatitis C virus (HCV), Sendai and Coxsackie B viruses, and induces immune response to prevent further infection of the virus (Heil et al., 2004; Zhang et al., 2016).

TLR7 can be activated directly or indirectly by a number of nucleotide analogues, such as 2′-deoxyguanosine (dG), 8-hydroxydeoxyguanosine (8-OHDG), 8-HYDROXYguanosine (8-OHG) which is capable to activate TLR7 and its downstream IFN significantly when synergistically binds poly U ssRNA as a therapeutic agent (Shibata et al., 2016; Davenne et al., 2020). Isatoribine (Lee et al., 2003), 7-deazaguanosine (Lee et al., 2003), and loxoribine (Dzopalic et al., 2010) all are known Guanosine (G) analogs that can activate TLR7 and induce downstream immune responses to inhibit virus infection (Horsmans et al., 2005). In addition to the above, polycyclic organic molecules are also a type of TLR7 agonists which principally contain imiquimod (R837) (Dockrell and Kinghorn, 2001), resiquimod (R848) (Meyer et al., 2013), 3 M-011, and PF-04878691 (Fidock et al., 2011). These molecules also act as TLR7 agonists to stimulate immune cells to produce interferons and interleukins to fight viral infections (Dockrell and Kinghorn, 2001; Garland, 2003).

TLR7/8 agonists can be used as vaccine adjuvants in combination with other components by activating TLR7/8 in cells and triggering a powerful immune response like the TLR4 agonists mentioned above (Tiboni et al., 2021). Several small molecule TLR7/8 agonists also have obtained approval of clinical or studied, such as resiquimod which transiently reduced viral levels but had some side effects interfering with the clinical effect (Pockros et al., 2007).

### TLR9 agonists

TLR9 is located on the endosomal membrane of cells (Kawasaki and Kawai, 2014). Based on TLR 9-binding ligands, an agonist that activates TLR9 needs to be CpG-rich (Agrawal and Kandimalla, 2007). There are many kinds of synthetic nucleotides designed according to TLR9 binding properties, all of which can activate TLR9 expression to varying degrees (Patinote et al., 2020).

Accordingly, current TLR9 agonists probably include multiple categories like SAR-21609, AVE0675, and SD-101 (Hennessy et al., 2010; Keogh and Parker, 2011); the synthetic oligonucleotide agatolimod (Hennessy et al., 2010; Wang et al., 2013); AZD1419 (Keogh and Parker, 2011; Psallidas et al., 2021) (IMO)-2055, IMO-2125, QAX-935 (IMO-2134), DIMS0150 (Hennessy et al., 2010), a synthetic molecule comprised of the nucleotides MGN-1703 and MGN-1706 (Hennessy et al., 2010). Several synthetic CpG oligonucleotide sequences have been tentatively identified as candidate investigational vaccine adjuvants due to their efficacy and safety, and CpG 1018 has been tested as a human vaccine adjuvant (Bode et al., 2011; Pulendran et al., 2021). CpG can be used to synthesize a variety of TLR9 agonists, which, by binding to TLR9, partly trigger the body’s cellular immune response to kill intracellular antigens more effectively (Crotty, 2019).

A team of scientists from the United States recently developed a formulation of a TLR9 agonist and aluminum hydroxide (AH: CpG preparation) to increase the immunogenicity of the S protein RBD of SARS-CoV-2. The results showed that RBD-based vaccines containing the adjuvant induced strong anti-SARS-CoV-2 neutralization immunity in both young and elderly mice. Further validation is needed. Another study reported a method of replacing mRNA vaccines using electric-changing delivery carriers that release transporters (CARTs) (Haabeth et al., 2021). The researchers customized vaccine immunogenicity by adding native non-immunogenic vectors to co-adjuvants with CpG motif (CpG-ODN), such as oligodeoxynucleotides. Mice inoculated with mRNA-CART vaccine produced treatment-specific RBD neutralizing antibodies in the circulating and pulmonary bronchial fluid. In addition, mice vaccinated with the vaccine elicited a strong and durable RBD-specific Th1 T cell immune response, including CD4 T cell and CD8 T cell memory immune response.

### TLR7/8 antagonists

TLR7 and TLR8 antagonists have been studied and used for in the treatment of a variety of diseases. By inhibiting the expression of TLR7 and TLR8, they can reduce the excessive inflammatory response of patients in the middle and late stage of different diseases and improve the survival rate (Ghosh et al., 2006). According to the binding preference of TLRs ligands and the conformational changes of receptors and ligands, various synthetic molecules that can inhibit the activity of TLRs have been designed as antagonists of TLRs. Some of these antagonists can simultaneously target multiple TLRs, while others specifically target one TLRs. Different antagonists can be selected according to different conditions of disease treatment and research.

The most important TLR8 antagonists contain pyrazolo [1,5-α]pyrimidine (Zhang et al., 2018), CU-CPT8m (Zhang et al., 2018) and VTX-763 (Wu et al., 2015). CU-CPT8m is designed to bind to the hydrophobic pocket in the case of TLR8-TLR8 dimer, thereby controlling changes in TLR8 imagination to inhibit TLR8 activity (Zhang et al., 2018).

Specific antagonists targeting TLR7 are CPD-6 and CPD-7 (Tojo et al., 2020), and there are also antagonists DV-1179 and IMO-3100 that simultaneously target TLR7/9. The antagonist IMO-8400 can simultaneously target the activity of TLR7/8/9 and significantly inhibit the inflammatory response in the process of pathogen infection (Gao et al., 2017). The above-mentioned antagonists against different TLRs can inhibit the excessive expression of inflammatory factors in the later stages of various diseases and thus reduce the inflammatory response. This has implications for the current treatment of patients who have progressed to late COVID-19, which could be combined with safe doses of TLRs antagonists and other clinical anti-inflammatory drugs to prevent cytokine storms.

### TLR9 agonists

Various antagonists of TLR9 have been proposed as an inhibitory target to suppress excessive inflammation and thrombotic complications in patients with advanced COVID-19 (Bezemer and Garssen, 2020). TLR7/9 antagonists known to be widely used, such as chloroquine (CQ) and hydroxychloroquine (HCQ) which are clinically applied to treat immune-mediated inflammatory diseases as the antimalarial drugs. Hydroxychloroquine works by inhibiting the binding of TLR9 to the ligand CPG-DNA so that TLR9 cannot be activated (Rutz et al., 2004). It has previously been suggested as a clinical treatment for COVID-19, but subsequent trials have had poor results and failed to actually reduce mortality in patients with COVID-19 (Chen et al., 2021).

**FIGURE 2:**
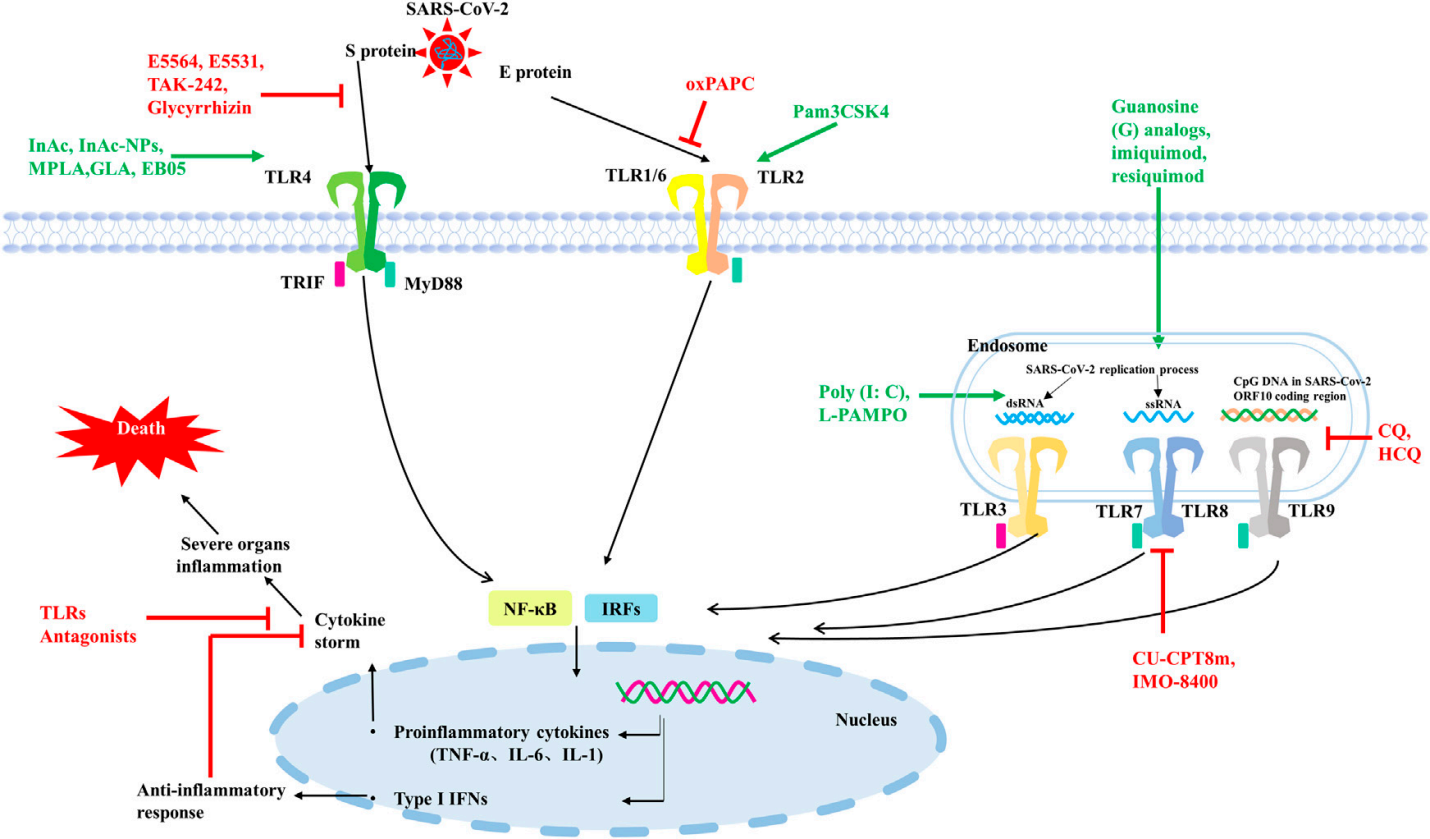
Some TLRs may be involved in the recognition process of SARS-CoV-2. TLR2 on the cell surface mainly senses SARS-CoV-2 E protein, TLR4 mainly recognizes SARS-CoV-2 S protein. TLR3 recognizes dsRNA produced during SARS-CoV-2 replication, TLR7/8 senses ssRNA of SARS-CoV-2, and TLR9 recognizes CPG-containing sequences of the SARS-CoV-2 genome. These TLRs recognize SARS-CoV-2 and activate downstream signaling pathways, triggering downstream inflammatory factors to participate in the immune response. TLRs agonists are shown in green and TLRs antagonists are shown in red.

In conclusion, both TLRs on cell membrane and TLRs on cell endosomal membrane may be involved in the invasion and infection process of SARS-CoV-2, and different TLRs are activated by recognizing different elements on SARS-CoV-2. Further activation of downstream immune signaling pathways, triggering different types of interferon, interleukins such as IFNα, β and IL-6 (Figure 2).

## Discussion

Since 2020, COVID-19 has continued to ravage the world, seriously affecting human health and causing death. Even after recovery, many diseases still occur. At present, the main method of human prevention is still vaccination, and different types of vaccines from multiple biological companies have different preventive effects and side effects. Vaccination around the world has limited the development of COVID-19 to some extent, but vaccination alone cannot significantly cut off the transmission path and speed of COVID-19 due to differences in the duration, quality and completeness of vaccination, and even differences in the prevention measures in place among the population of each country. It is known that SARS-CoV-2 can invade multiple organs of the body through ACE2, such as lung, heart, kidney and other organs, causing inflammation and serious organ failure and even death. However, some studies have shown that SARS-CoV-2 not only invades cells through ACE2, but also binds to individual TLRs and triggers the release of a series of proinflammatory factors or type I IFNs in innate immunity. Sars-cov-2 mainly activates TLR2, TLR3, TLR4, TLR7/8 and TLR9, and induces corresponding downstream response pathways. Type I IFNs have antiviral effect in the early stage of infection, while excessive accumulation of proinflammatory cytokines will cause immune damage and organ failure. In severe cases of SARS-CoV-2 infection, cytokine release syndrome is a significant hallmark. Therefore, some TLRs agonists, mentioned in Figure 2; Table 1, can be used in the early stage of infection or as vaccine adjuvants to induce the production of type I IFNs to limit SARS-CoV-2 infection, or to activate the release of appropriate proinflammatory cytokines to promote adaptive immunity. In patients with severe disease or complications who have already developed cytokine storm, partial TLRs antagonists, as shown in Figure 2 and Table 1, can be used in combination with conventional treatment to control inflammation production and mitigate viral infection. The most countries are not immune to COVID-19, so there are still a large number of COVID-19 patients who need medication for clinical treatment. On the basis of current therapeutic drugs, TLRs regulatory molecules can be assisted in collaborative drug delivery, which is expected to achieve better therapeutic effects at different stages of COVID-19.

**TABLE 1:** TLRs involved in recognition of SARS-CoV-2.

TLRs	Primary Localization	Ligands	Adaptor Molecules	Agonists	Antagonists	Potential drugs known to have an effect against SARS-CoV-2 infection
TLR2	Cell surface	Triacyl lipopeptides, Diacyl lipopeptides, Bacterial lipoproteins	MyD88	Pam3CSK4, CU-T12-9, BLP, L-PAMPO	oxPAPC	• In the early stages of infection, TLRs agonists such as Pam3CSK4, Poly (I: C), L-PAMPO, Imiquimod (R837), Resiquimod (R848) can promote the body to produce type I IFNs and appropriate amount of cytokines to inhibit the further infection of SARS-CoV-2. At the same time, TLRs agonists can be used as vaccine adjuvants to enhance the immune effect
TLR3	Endosome	dsRNA	TRIF	CU-CPT17e, IPH-3102, Rintatolimod, Poly (I: C), L-PAMPO	CU-CPT4a	
TLR4	Cell surface	LPS, Viral envelope Glycoproteins; etc	MyD88/TRIF	InAc, InAc-NPs, MPLA, GLA, EB05	RsDPLA, Eritoran (E5564), E5531, Resatorvid (TAK-242), Nifuroxazide, Curcumin, Turmeric, Glycyrrhizin	
TLR7	Endosome	ssRNA, Imidazoquinolines, Guanosine analogs	MyD88	Isatoribine, 8-OHdG, 8-OHG, SM-360320, CL264, SM-324405, Imiquimod (R837), Resiquimod (R848), 3 M-011, PF-04878691, Vesatolimod (GS-9620), AZD8848	CPD-6, CPD-7, DV-1179, IMO-3100, IMO-8400	• In the treatment phase of severe patients, severe inflammatory response is often accompanied by the persistent release of proinflammatory cytokines. Since TLRs are also involved in the activation of innate immunity during SARS-CoV-2 infection, cytokine storm will be generated. Therefore, TLRs antagonists such as oxPAPC, Eritoran (E5564), CQ, and HCQ can reduce the concurrent inflammation caused by viral infections. In particular, combined use of antagonists that block activation of multiple TLRs, such as IMO-8400, is recommended
TLR8	Endosome	ssRNA, Imidazoquinolines	MyD88	VTX-1463, VTX-2337 (motolimod), Selgantolimod (GS-9688)	CU-CPT8m, VTX-763, CU-CPT9a, CU-CPT9b, IMO-8400	
TLR9	Endosome	CpG DNA	MyD88	AZD1419, IMO-2055, MGN-1703, MGN-1706, IMO-2125, QAX-935 (IMO-2134), ISS1018	DV-1179, IMO-3100, IMO-8400, CQ, HCQ	

There are currently no regulatory TLRs drugs approved for clinical use due to the lack of follow-up scientific studies on efficacy and safety or the lack of effective standard control treatments for COVID-19. In addition, the presence of severity of COVID-19 in many study patients complicates the understanding of the pathophysiology and pharmacodynamics of the disease, and subsequent studies of regulatory TLRs drugs should also pay attention to the dual role of TLRs in the progression of COVID-19 disease. As mentioned above, several TLRs antagonists, such as CQ and HCQ, as the result of having not achieved the expected improvement of survival indicators in clinical trials of COVID-19 treatment, they are no longer being suggested as candidate treatments for COVID-19 although they have anti-malaria and anti-virus effects. Therefore, whether multiple agonists and antagonists of TLRs can be approved as future COVID-19 treatment drugs still needs long-term clinical trials to ensure reasonable dosage and actual effect in combination therapy.
